# The miR-1269a/PCDHGA9/CXCR4/β-catenin pathway promotes colorectal cancer invasion and metastasis

**DOI:** 10.1186/s11658-024-00656-9

**Published:** 2024-11-26

**Authors:** Haitao Mei, Qingshan Luo, Junyong Weng, Jialing Hao, Jinfeng Cai, Runkai Zhou, Ce Bian, Yingzi Ye, Shengzheng Luo, Yugang Wen

**Affiliations:** 1 Department of Gastrointestinal Surgery, Shanghai Municipal Hospital of Traditional Chinese Medicine, Shanghai University of Traditional Chinese Medicine, 274 Zhijiang Middle Road, Shanghai, 200071 China; 2 Department of General Surgery, Shanghai General Hospital, Shanghai Jiao Tong University School of Medicine, 85 Wujin Road, Shanghai, 200080 China; 3 Department of Colorectal Surgery, Changzheng Hospital, Navy Medical University, 415 Fengyang Road, Shanghai, 200003 China; 4 Department of Gastroenterology, Shanghai General Hospital, Shanghai Jiao Tong University School of Medicine, 85 Wujin Road, Shanghai, 200080 China; 5 Department of Colorectal Surgery, Fudan University Shanghai Cancer Center, 270 Dong’an Road, Shanghai, 200032 China; 6 Department of Infectious Diseases, Children’s Hospital of Fudan University, 399 Wanyuan Road, Shanghai, 201102 China

**Keywords:** CRC, PCDHGA9, miR-1269a, HOXA1, CXCR4, β-Catenin

## Abstract

**Background:**

Colorectal cancer (CRC) is the third most common cancer worldwide and the second leading cause of cancer-related death. This research focuses on investigating the impact and underlying molecular mechanisms of protocadherin gamma subfamily A, 9 (PCDHGA9) on the invasion and metastasis of CRC, aiming to identify more precise molecular markers for the diagnosis and prognosis of CRC.

**Methods:**

PCDHGA9 expression was detected using quantitative real-time quantitative polymerase chain reaction (RT-qPCR) in 63 pairs of colorectal cancer tissues. Differential gene expression from high-throughput sequencing was analyzed using ingenuity pathway analysis (IPA) to explore the biological functions of PCDHGA9 and its potential regulated genes. Bioinformatics tools were employed to explore potential upstream regulatory microRNAs of PCDHGA9. Dual-luciferase assays were performed to demonstrate the regulation between PCDHGA9 and miR-1269a. Protein mass spectrometry suggested an interaction between PCDHGA9 and HOXA1. JASPAR predicted that HOXA1 may act as a transcription factor of CXCR4. Coimmunoprecipitation, dual-luciferase assays, and nuclear–cytoplasmic fractionation experiments confirmed the molecular mechanism involving PCDHGA9, CXCR4, HOXA1, and β-catenin. Transwell, wound healing, and western blot assays were conducted to confirm the impact of PCDHGA9, miR-1269a, and CXCR4 on the invasion, metastasis, and epithelial–mesenchymal transition (EMT) functions of CRC cells in in vitro experiments. A whole-body fluorescence imaging system was used to evaluate the combined impact of miR-1269a and PCDHGA9 on the invasion and metastasis of CRC in in vivo experiments.

**Results:**

The expression of PCDHGA9 was found to be lower in CRC tissues compared with their corresponding adjacent tissues. Low expression of PCDHGA9 potentially correlated with worse prognosis and increased chances of invasion and metastasis in CRC. miR-1269a was highly expressed in CRC tissues and acted as a negative regulator for PCDHGA9, promoting invasion, migration, and EMT of CRC cells. PCDHGA9’s interaction with HOXA1 downregulated CXCR4, a transcription factor, leading to accumulation of β-catenin and further promoting invasion, migration, and EMT of CRC cells.

**Conclusions:**

PCDHGA9, acting as a tumor suppressor, is downregulated by miR-1269a. The low level of PCDHGA9 activates the Wnt/β-catenin pathway by releasing its interaction with HOXA1, promoting the expression of CXCR4, and causing invasion, migration, and EMT in CRC.

**Graphical Abstract:**

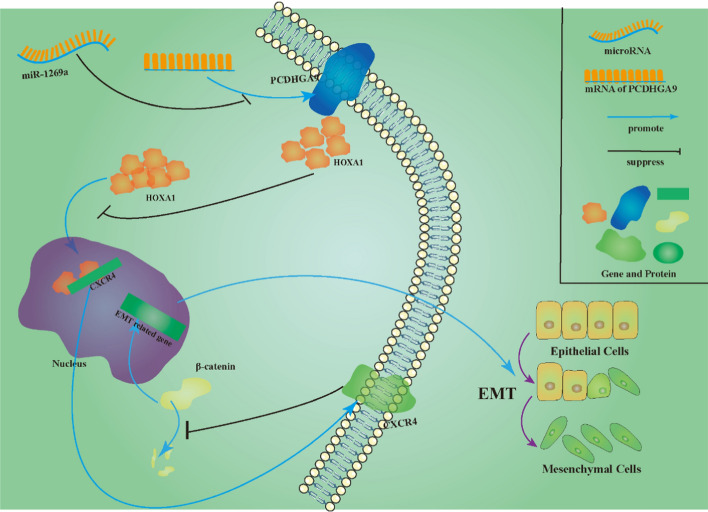

**Supplementary Information:**

The online version contains supplementary material available at 10.1186/s11658-024-00656-9.

## Background

According to Global Cancer Statistics 2020, colorectal cancer (CRC) ranks as the third most common cancer worldwide, accounting for an estimated 19.3 million new cancer cases, and is the second leading cause of cancer-related death, contributing to approximately 10.0 million cancer-related fatalities. The situation is further exacerbated by the projection that CRC mortality rates will escalate with the anticipated rise in cancer incidences by 2040 [1]. Despite the implementation of advanced diagnostic and therapeutic methodologies, early-stage CRC demonstrates favorable 5-year survival rates following radical resection surgery [2]. However, among new diagnoses, 20% of patients have metastatic disease at primary diagnosis and another 25% who present with localized disease will later develop metastases [3]. Patients with advanced CRC have a 5-year survival rate of less than 5% after surgical treatment, chemotherapy, and immunotherapy, and 50% of them die from subsequent metastatic disease [4]. Particularly in the case of CRC patients with distant organ metastases, the opportunity for curative surgical intervention becomes unattainable, and without treatment, these patients have a median survival time of only 6–9 months, while if treated and responding to optimal chemotherapy, they can potentially live for a median of 13–18 months [5, 6]. Thus, to further improve treatment outcomes and extend patient survival, it is important to identify the underlying mechanism of CRC invasion and metastasis to identify more prominent targets.

Tumor invasion and metastasis arise from a complex interplay of genetic alterations and consequential shifts in cellular traits, among which the epithelial–mesenchymal transition (EMT) is a prominent contributor to their invasive and metastatic behaviors [7, 8]. EMT of tumor cells is a process whereby tumor cells gradually lose their epithelial characteristics and transform into mesenchymal cells. This transformation involves a decrease in the expression of E-cadherin and EpCAM and a corresponding increase in of N-cadherin, vimentin, Snail, and Slug expression, causing alterations in cellular structure that enable tumor cells to invade, metastasize, and become resistant to therapeutic interventions [9, 10].

Numerous studies have provided compelling evidence regarding the crucial role of the Wnt pathway in EMT [11–15]. The Wnt signaling pathway constitutes a multifaceted network of protein interactions, primarily recognized for its involvement in embryonic development and important physiological processes during adulthood [16]. However, it also plays an important role in tumorigenesis, cancer cell invasion, metastasis, and drug resistance [17]. β-Catenin, a pivotal player in the Wnt signaling pathway, is also a crucial participant in the EMT process. Typically, in the absence of Wnt ligands, cytoplasmic β-catenin undergoes phosphorylation by protein complexes (i.e., AXIN, GSK3β, CK1, and APC), following which it is ubiquitinated and subsequently subjected to proteasomal degradation [18]. Conversely, in the presence of Wnt ligands, cytoplasmic β-catenin translocates to the nucleus, and its accumulation in the nucleus enhances its interaction with T-cell-specific factor (TCF) and lymphoid enhancer-binding factor (LEF), leading to the upregulation of TCF/LEF target genes [19, 20].

In our previous study, we found that PCDHGA9 expression was reduced in gastric cancer tissues compared with corresponding paraneoplastic tissues [21]. It also interacted with β-catenin to affect the location of β-catenin in gastric cancer cells to inhibit the Wnt pathway, EMT, invasion, and metastasis of gastric cancer cells [22]. However, in CRC, we found that PCDHGA9 affected β-catenin in another way to inhibit the Wnt pathway, EMT in colorectal cancer cells, and CRC invasion and metastasis. In this study, we examined the expression of PCDHGA9 in CRC tissues and found that PCDHGA9 expression was reduced in CRC compared with paraneoplastic tissues and correlated with tumor differentiation, staging, and invasion. Furthermore, we investigated the role of PCDHGA9 in reducing the invasive and metastatic tendencies of CRC, explored miR-1269a as an explanatory factor for the diminished PCDHGA9 levels in CRC, and revealed the intricate interplay among the constituents of the miR-1269a/PCDHGA9/CXCR4/β-catenin axis, delineating their roles and cumulative effects on the invasive and metastatic functions of CRC.

## Methods

### Patients and tissue specimens

We randomly selected 63 paired colorectal cancer tissue samples (including cancer and adjacent normal tissues) from the specimen library of patients who underwent radical surgery at Shanghai General Hospital between 2013 and 2014. The cohort comprised 35 males and 28 females, ranging in age from 35 to 84 years, with a median age of 65 years. Inclusion criteria were: (1) absence of preoperative chemotherapy or neoadjuvant therapy for all patients, (2) postoperative pathological confirmation of colorectal cancer by two pathology experts, and (3) successful completion of radical surgery. Exclusion criteria included: (1) patient age under 25 years, (2) preoperative chemotherapy or neoadjuvant therapy, (3) concurrent presence of other tumor types or a history of tumors, (4) family history of hereditary colorectal cancer or other related genetic disorders, and (5) inability to undergo radical surgery. This study was approved by the Ethics Committee of Shanghai General Hospital, and all patients provided informed consent.

All the tissues specimens we used for PCR were collected from freshly resected colorectal cancer specimens within 30 min and then stored at −80 °C.

### Bioinformatic tools

We used starBase (https://starbase.sysu.edu.cn/starbase2/), miRBD (http://www.mirdb.org/miRDB/policy.html), Targetscan (http://www.targetscan.org/vert_71/), and JASPAR (https://jaspar.genereg.net/).

### Quantitative real-time polymerase chain reaction (qRT-PCR)

Total RNA extraction from cells and tissues was performed by using the RNA-Quick Purification Kit (Shanghai Yishan), following the manufacturer's instructions, and HyperScript™ III first-strand cDNA synthesis kit with gDNA Remover (NovaBio) was used for reverse transcription. qRT-PCR was performed using SYBR Green premix PCR (NovaBio). All primers are listed in Table 1. GAPDH and U6 were used as reference genes for mRNA and microRNA genes to calculate the relative gene expression levels (2^−ΔΔCT^). The gene of each sample was run in triplicate.

**Table 1:** Information on gene primers

Gene	Forward	Reverse
PCDHGA9	AAGTCCTGGTTGAAGACAGAGTG	CTTCTGGAAGTGGATAACGTGC
GAPDH	GTCTCCTCTGACTTCAACAGCG	ACCACCCTGTTGCTGTAGCCAA
CXCR4	CTCCTCTTTGTCATCACGCTTCC	GGATGAGGACACTGCTGTAGAG
miR-1269a	CGCTGGACTGAGCCGTGC	AGTGCAGGGTCCGAGGTATT
miR-3179	CGCGAGAAGGGGTGAAATTT	AGTGCAGGGTCCGAGGTATT
U6	GCTCGCTTCGGCAGCACATATAC	AGTGCAGGGTCCGAGGTATT

### Western blot

Total protein was extracted according to the manufacturer’s instructions for RIPA Lysis Buffer (Beyotime, Jiangsu, China). The separation of cytoplasmic and nuclear proteins was conducted based on the instructions of the Nuclear and Cytoplasmic Protein Extraction Kit (Beyotime, Jiangsu, China). Protein concentrations were quantified using the BCA Protein Assay Kit (Beyotime, Biotechnology, Jiangsu, China). An equivalent amount of protein, mixed with loading buffer, was loaded onto SDS-PAGE gels (Epizyme, Shanghai, China) for electrophoresis and subsequent transfer onto polyvinylidene difluoride membranes (Millipore, Billerica, USA). The membranes were blocked using Protein Free Rapid Blocking Buffer (Epizyme, Shanghai, China) at room temperature for 15 min, followed by incubation with specific primary antibodies at 4 °C for 8 h. Subsequently, membranes were washed thrice with TBST buffer for 5 min each. Horseradish peroxidase-labeled secondary antibodies were combined with the membranes at room temperature for 1 h and detected using an ECL kit. The obtained images were analyzed using ImageJ 1.43 software. GAPDH was used as a control for whole-cell lysates. Information on all the antibodies is listed in Table 2.

**Table 2:** Information on the antibodies

Primary antibody	Dilution ratio	Company
Anti-PCDHGA9	1:1000	Abnova
Anti-GAPDH	1:1000	Cell Signaling Technology
Anti-β-catenin	1:1000	Cell Signaling Technology
Anti-E-cadherin	1:1000	Cell Signaling Technology
Anti-N-cadherin	1:1000	Cell Signaling Technology
Anti-vimentin	1:1000	Cell Signaling Technology
Anti-Snail	1:1000	Cell Signaling Technology
Anti-CXCR4	1:500	Abclonal
Anti-HOXA1	1:500	Affinity
Anti-Flag	1:1000	Sigma
Anti-LaminA	1:1000	Cell Signaling Technology
Anti-β-tubulin	1:1000	Cell Signaling Technology
Second antibodies		
Goat anti-mouse IgG (H+L)	1:10,000	Jackson
Mouse anti-rabbit IgG (conformation specific) mAb	1:1000	Cell Signaling Technology

### Cell culture, transfection, and screening of stable cell lines

CRC cell lines HCT116 (TCHu 99), RKO (TCHu116), HCT8 (TCHu18), SW620 (TCHu101), and human embryonic kidney cell line 293T (SCSP-502) were purchased from the National Collection of Authenticated Cell Cultures, Chinese Academy of Sciences (Shanghai, China) and cultured in DMEM medium (Gibco, California, USA) supplemented with 10% fetal bovine serum (FBS) (Gibco, CA, USA) in a humidified incubator maintained at 37 °C with 5% CO_2_. There are several considerations for selecting HCT116, RKO, HCT8, and SW620 as the experimental cell lines: HCT116 is our preferred cell line owing to its mutated KRAS gene, specifically in codon 12 or 13 of exon 2, which leads to the activation of the RAS gene. Its downstream Wnt pathway is related to PCDHGA9. Additionally, HCT116 exhibits good tumorigenicity, facilitating in vivo experiments. HCT8, a rectal-derived cell line, shares similar KRAS mutation patterns with HCT116, serving as a supplement to ensure the reliability of PCDHGA9 studies in colorectal cancer. RKO expresses wild-type P53, which, like PCDHGA9, is a tumor suppressor gene. SW620, a cell line derived from metastatic tumors, commonly exhibits KRAS mutations. All cell information can be referenced at https://www.cellbank.org.cn/.

Transfections of inhibitors, mimics, and other plasmids were performed using Lipofectamine 2000 (Invitrogen), adhering to the manufacturer’s guidelines. The stably expressing PCDGA9 cell line was screened using puromycin based on the total 24-h minimum lethal concentration of each cell line. For the cotransfected PCDHGA9 and miR-1269a cells, puromycin and blasticidin (Invivogen) were used for screening. Detailed information regarding all plasmids is provided in Table 3.

**Table 3:** Information on all plasmids

Plasmid	Company
PCDHGA9 vector (V_PCDHGA9_) (pLenti-CMV-MCS-3FLAG-PGK-Puro)	OBiO
PCDHGA9 OE (OE_PCDHGA9_) (pLenti-CMV- PCDHGA9-3FLAG-PGK-Puro)	OBiO
PCDHGA9 NC (Plvx-NC)	OBiO
PCDHGA9 Sh (Plvx-shRNA1, Target sequence: CCCAAATTCTTGACCGAGAAA) PCDHGA9 Sh2 (Plvx-shRNA2, Target sequence: GCGGAAGATTAGTCCTGCTAT) PCDHGA9 Sh3 (Plvx-shRNA3, target sequence: CCTGCAAGTGACTGACATCAA)	OBiO
CXCR4 vector (pLenti-CMV-MCS-3FLAG-PGK-Puro)	OBiO
CXCR4 OE (pLenti-CMV-CXCR4-3FLAG-PGK-Puro)	OBiO
HOXA1 vector (pLenti-CMV- MCS-6HIS-PGK-Puro)	OBiO
HOXA1 OE (pLenti-CMV-HXA1-6HIS-PGK-Puro)	OBiO
PCDHGA9-WT- miR-1269a (GP-miRGLO, ctaacaggac caatggatta aactggcatt tcagtccaag gaagctcgaa gcaggtttag PCDHGA9-MUT- miR-1269a (GP-miRGLO, ctaacaggac caatggatta aactgCGTtt tGTCAGGTag gaagctcgaa gcaggtttag)	Genepharma
CXCR4 WT (pGL4.10-CXCR4 promoter (WT), chr2:136118150–136120149)	OBiO
CXCR4 MUT1 (pGL4.10-CXCR4 promoter (MUT1), chr2:136118150–136119049)	OBiO
CXCR4 MUT2 (pGL4.10-CXCR4 promoter (MUT2), chr2:136118150–136118884)	OBiO
miR-1269a vector (V_miR-1269a_) (pSLenti-EF1- Luc2-P2A-BSR-CMV-MCS-WPRE)	OBiO
miR-1269a OE (OE_miR-1269a_) (pSLenti-EF1- Luc2-P2A-BSR-CMV- miR-1269a-WPRE)	OBiO

### Transwell and wound healing

Cell invasion was tested using Transwell chambers (Corning, NY, USA), where 1 × 10^5^ cells, 200 μl of serum-free medium, and Matrigel were placed in the Transwell chamber, and 600 μl of 10% FBS medium was added to a 24-well plate. After 24 h, the lower side of the Transwell was fixed with methyl alcohol and stained with 0.1% crystal violet, and invasive cells were counted under a microscope. Migration ability was assessed through a wound healing assay using a six-well plate. Cells were cultured until adhered, and a scratch was made. Serum-free medium was added, and the scratch width was measured at 0 and 48 h to evaluate migration ability. Each experiment was done in triplicate.

### High-throughput sequencing and ingenuity pathway analysis

Stable transfected vector or PCDHGA9-overexpressing plasmids were screened in HCT116 cells using puromycin. High-throughput sequencing was performed on three samples from each group, except for one outlier pair. The differential genes were analyzed in the remaining two pairs of samples. Ingenuity pathway analysis was conducted using IPA software from the Library of Shanghai Jiaotong University of Medicine (Shanghai, China).

### Dual luciferase assay

To test the regulatory relationship between PCDHGA9 and miR-1269a, wild-type PCDHGA9 promoter reporter plasmid or mutant PCDHGA9 promoter reporter plasmid (GenePharma, Shanghai, China) were cotransfected into 293T cells along with either NC or miR-1269a mimic. For testing the regulatory relationship between CXCR4 and HOXA1, wild-type (WT), MUT1, or MUT2 CXCR4 promoter reporter plasmids (OBiO, Shanghai, China) were transfected into 293T cells along with vector or overexpressing HOXA1 plasmids. After 24 h of incubation, luciferase activity was measured following the manufacturer’s instructions using the Dual-Luciferase Assay Kit (Promega, Beijing, China).

### Coimmunoprecipitation (Co-IP) and protein mass spectrometry

Protein samples from vector or overexpressing PCDHGA9 cells were lysed using a precipitate lysis buffer. A portion of these protein samples was utilized as input samples, while another portion was mixed with anti-Flag antibody and incubated at 4 °C for 2 h. Then, Protein A/G PLUS-Agarose beads (MedChemExpress, Shanghai, China) were added and incubated at 4 °C for 12 h. After incubation, the beads from these two groups were washed using a wash buffer to prepare the immunoprecipitation (IP) samples. Subsequently, all four samples were subjected to western blotting analysis. Additionally, the IP samples from the group overexpressing PCDHGA9 were further processed for protein mass spectrometry (Bioprofile, Shanghai, China).

### Nude mice metastatic models and immunohistochemical staining

Four groups of HCT116 cells, cotransfected with vector of PCDHGA9 (V_PCDHGA9_) or overexpression of PCDHGA9 (OE_PCDHGA9_) and vector of miR-1269a (V_miR-1269a_) or overexpression of miR-1269a (OE_miR-1269a_), were prepared to establish an in vivo metastasis model. Male BALB/C nude mice at 4 weeks of age were injected with 1 × 10^6^ cells for each group (*n* = 5) via the tail vein. The mice were housed in a specific pathogen-free animal room for 4 weeks. Whole-body fluorescence images were captured using a live animal imaging instrument (Caliper Life Sciences, Hopkinton, MA, USA) after intraperitoneal injection of d-luciferin potassium salt (CSNpharm, Chicago, IL, USA). Subsequently, the mice were sacrificed to examine lung metastatic tumors, which were subjected to Immunohistochemical staining (BiosBiological, Wuhan, China).

### Statistical analysis

The SPSS 22.0 statistical software package (SPSS, Chicago, IL, USA) was employed for data analysis. Quantitative results were compared using the Student’s *t*-test. Survival curves were generated using the Kaplan–Meier method, and differences in survival rates between PCDHGA9 high expression and PCDHGA9 low expression were assessed using the log-rank test. The relationship between the expression levels of PCDHGA9 and miR-1269a was evaluated using Pearson’s correlation. Statistical significance was defined for *P*-values less than 0.05.

## Results

### PCDHGA9 is downregulated in CRC and associated with poor prognosis

**Fig. 1:**
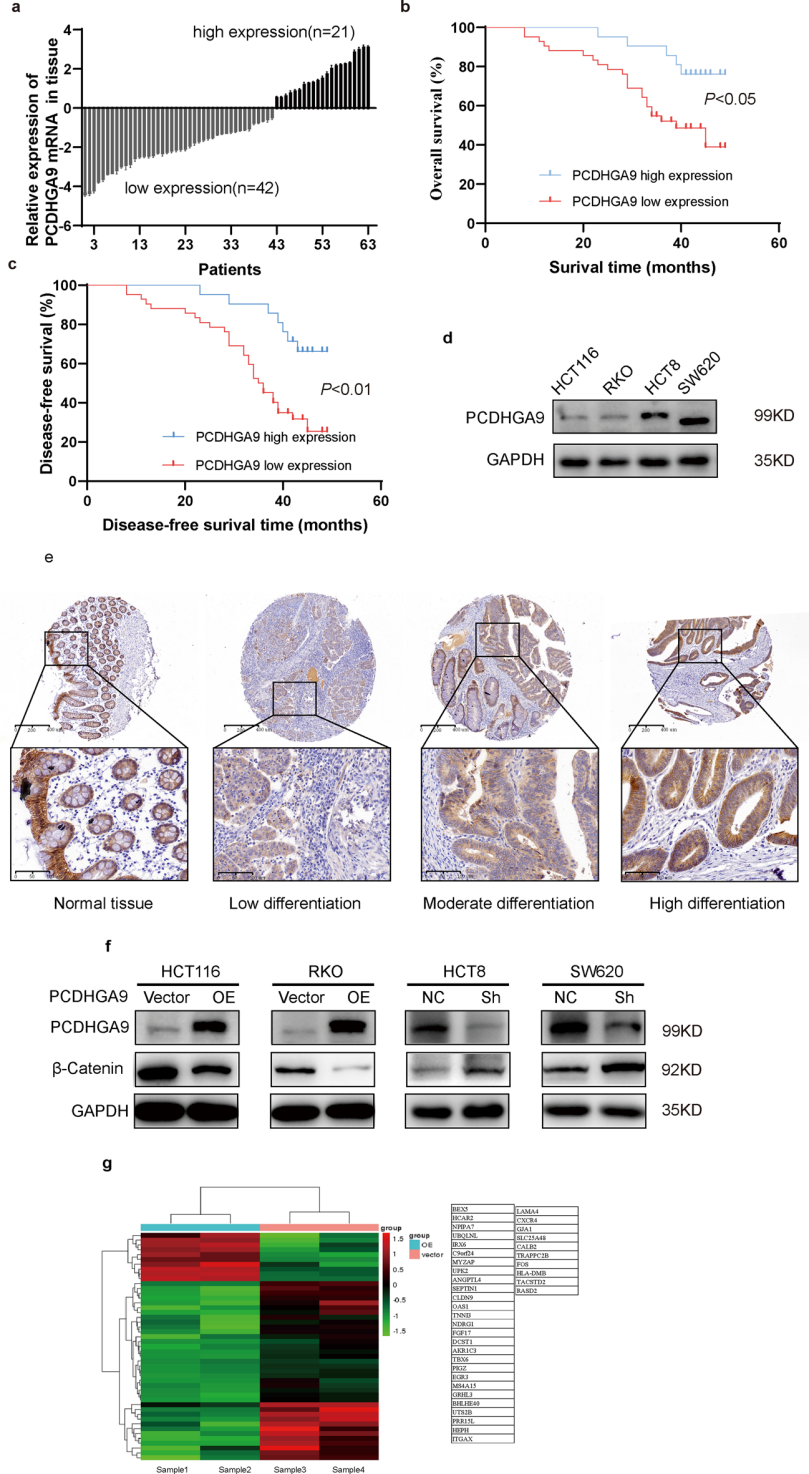
Comprehensive investigation of PCDHGA9 in CRC: expression in tissue, survival analysis, cell line manipulation, and high-throughput sequencing results. **a** Real-time quantitative PCR (qPCR) revealed decreased PCDHGA9 expression in 42 CRC tissues among 63 paired samples, compared with adjacent normal tissues. **b**, **c** Patients with low PCDHGA9 expression exhibited shorter overall survival and disease-free survival rates. **d**, **f** Western blot analysis assessed PCDHGA9 expression in HCT116, RKO, HCT8, and SW620 cells, as well as the impact of overexpressing or knocking down PCDHGA9 on PCDHGA9 and β-catenin expression. **e** Immunohistochemistry exhibits the expression of PCDHGA9 in CRC tissues with low, moderate, and high differentiation. **g** High-throughput sequencing identified differential gene expression between vector and PCDHGA9-overexpressing cells, with downregulated genes listed on the right

To assess the expression of PCDHGA9 in colorectal cancer tissues, we selected 63 paired tissue specimens from colorectal cancer patients who had undergone radical surgery at Shanghai General Hospital between 2013 and 2014, which were then subjected to RNA extraction and subsequent qRT-PCR analysis. The qRT-PCR results indicated that, among the 63 colorectal cancer patient tissues, 42 patients exhibited lower mRNA expression of PCDHGA9 in CRC tissue compared with paracancerous tissue (Fig. 1a), and overall survival and disease-free survival analysis demonstrated that colorectal cancer patients with reduced PCDHGA9 expression had poorer survival rates (Fig. 1b, c). Furthermore, poorly differentiated CRC tissues exhibited lower expression of PCDHGA9 (Fig. 1e).

To further investigate the significance of PCDHGA9 in colorectal cancer, we collected clinicopathological information of CRC patients, which was then assessed in combination with PCDHGA9 expression, particularly in regards to patient age, gender, tumor location, tumor size, tumor stage, tumor differentiation, tumor invasion degree, and lymphatic vascular metastasis. The chi-squared test showed that patient age, gender, tumor location, and tumor size were not related to PCDHG9 expression, while significant association was observed with patient tumor stage, tumor differentiation, tumor invasion degree, and lymphatic vascular metastasis (Table 4). Taken together, on the basis that these tumor characteristics, namely tumor stage, tumor differentiation, extent of tumor invasion, and lymphatic vascular metastasis, closely correspond to the inherent invasive and metastatic attributes of tumor cells, we speculated a correlation between PCDHGA9 and tumor invasion and metastasis.

**Table 4:** Correlation between PCDHGA9 expression and clinical information of patients (*n* = 63)

	Group	Low expression of PCDHGA9 (42)	High expression of PCDHGA9 (21)	*χ*^2^	*P*-value
Age (years)	< 65 (31)	20	11	0.127	0.722
	≥ 65 (32)	22	10		
Gender	Male (35)	22	13	0.514	0.473
	Female (28)	20	8		
Tumor location	Right hemicolon (25)	16	9	0.133	0.716
	Left hemicolon and retum (38)	26	12		
Tumor size	< 5 cm (53)	25	10	0.804	0.370
	≥ 5 cm (28)	17	11		
Stage of TNM	I + II (29)	15	14	5.399	0.020*
	III + IV (34)	27	7		
Differentiation	Low and moderate (50)	38	12	9.498	0.020*
	High (13)	4	9		
Invasion	To mucosa and submucosa layer (18)	6	12	12.60	0.001**
	To muscle and serous layer (45)	36	9		
Lymphatic vascular metastasis	No (44)	25	19	6.368	0.012*
	Yes (19)	17	2		

Bold represents significance: *P < 0.05 **P < 0.01 ***P < 0.001

### Downregulated PCDHGA9 promotes invasion, metastasis, and EMT of CRC cells

To verify this speculation, we detected the mRNA and protein expression of PCDHGA9 in four cell lines: HCT8, HCT116, RKO, and SW620. The results showed a relatively lower PCDHGA9 expression in HCT116 and RKO cells and higher expression in HCT8 and SW620 cells (Fig. 1d, Supplementary Fig. S1a). Subsequently, we employed lentiviral technology to construct an overexpressing PCDHGA9 cell line in HCT116 and RKO and a lentiviral vector containing short hairpin RNA targeting PCDHGA9 cell line in HCT8 and SW620 (Fig. 1f, Supplementary Fig. S1bc). Differential gene analysis was conducted between vector of PCDHGA9 and overexpression of PCDHGA9 in HCT116 using high-throughput sequencing (Fig. 1g). Clustering analysis of the differential genes using ingenuity pathway analysis (IPA) unveiled an association between PCDHGA9 and cell adhesion function (Supplementary Fig. S1e).

**Fig. 2:**
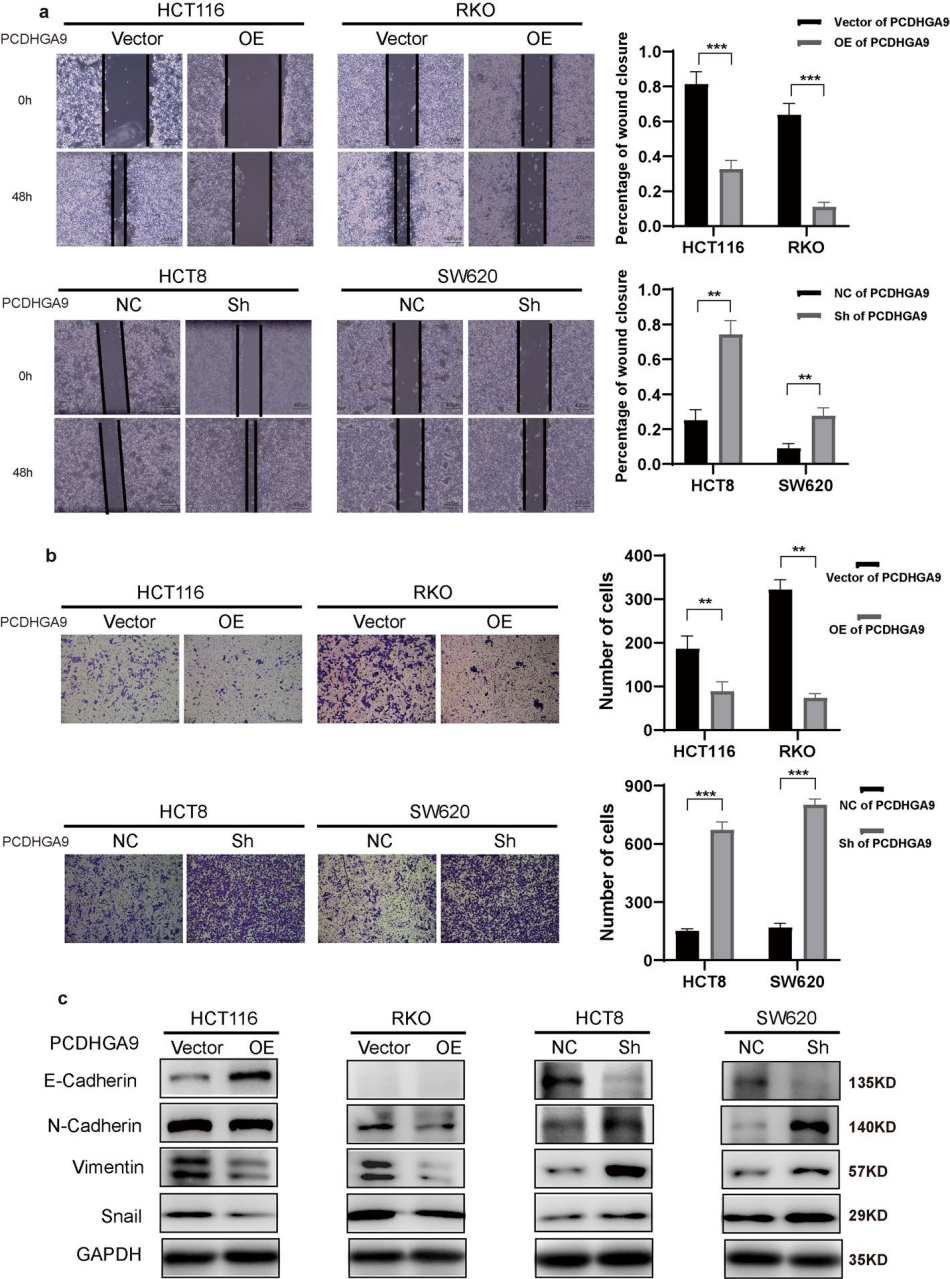
PCDHGA9 suppresses invasion, metastasis, and EMT of CRC cells. **a** Wound healing assays were conducted on CRC cells with overexpressed or downregulated PCDHGA9 and their respective controls. The width of wounds was measured at 0 and 48 h, and the percentage of wound closure was calculated and analyzed using the Student’s *t*-test. **b** Transwell assays were performed on CRC cells with manipulated PCDHGA9 expression and their controls, and the number of cells that had migrated through the chamber was observed and quantified. **c** Western blot analysis showing changes in E-cadherin, N-cadherin, vimentin, and Snail expression following the overexpression or downregulation of PCDHGA9. Notably, E-cadherin was not detected in RKO cells from our laboratory, resulting in the absence of an E-cadherin blot

Both the analysis of pathological data and sequencing results have confirmed a correlation between PCDHGA9 expression and the invasive and metastatic functions of CRC. Subsequently, we investigated the impact of PCDHGA9 on the invasion and metastasis abilities, along with the epithelial–mesenchymal transition (EMT) of CRC cells, through in vitro cellular assays. Wound healing and Transwell assays were performed to examine the influence on CRC cells’ migration and invasion abilities, and the results showed that knockdown of PCDHGA9 led to a faster scratch healing rate among CRC cells and an increased number of cells invading through the Transwell membrane compared with the negative control (NC) groups, while opposite results were observed in the overexpression of PCDHGA9 groups (Fig. 2a, b). Experiments involving assessment of the expression of EMT-related proteins showed that the knockdown of PCDHGA9 resulted in a significant decrease in E-cadherin levels and a significant increase in N-cadherin, vimentin, and Snail expression, while the overexpression of PCDHGA9 resulted in a significant increase in E-cadherin expression and significant reductions in N-cadherin, vimentin, and Snail levels (Fig. 2c).

Our previous investigation in gastric cancer revealed the inhibitory role of PCDHGA9 in the Wnt/β-catenin pathway, and here we extended similar research in colorectal cancer. We performed western blot (WB) to assess β-catenin protein levels and found a significant reduction in the intracellular β-catenin protein content in HCT116 and RKO cells following PCDHGA9 overexpression compared with the vector, and this pattern was reversed when PCDHGA9 was knocked down in HCT8 and SW620 cells (Fig. 1g). On the basis of these outcomes, we speculated that PCDHGA9 potentially reduces the invasion and metastasis of CRC by facilitating the degradation of β-catenin, thereby leading to a reduction in β-catenin protein present in CRC cells.

### miR-1269a downregulates PCDHGA9 and promotes invasion, metastasis, and EMT of CRC cells

**Fig. 3:**
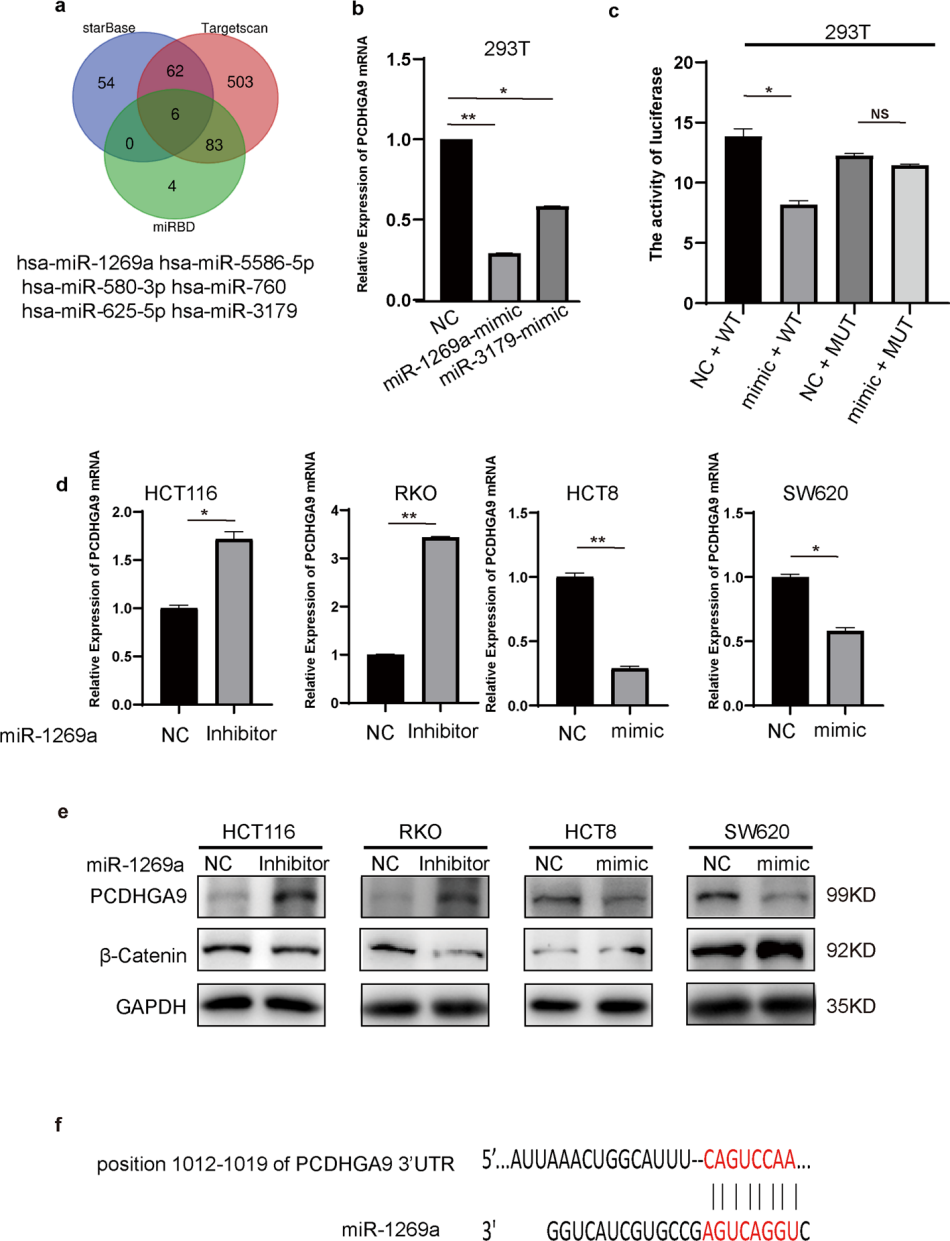
miR-1269a targets PCDHGA9 and downregulates PCDHGA9. **a** Venn diagram illustrating the microRNAs predicted to interact with PCDHGA9 across three databases (starBase, Targetscan, and miRBD). Among these, hsa-miR-1269a, hsa-miR-5586-5p, hsa-miR-580-3p, hsa-miR-760, hsa-miR-625-5p, and hsa-miR-3179 were identified as commonly predicted microRNAs. **b** PCDHGA9 expression was assessed via qPCR after transfection of miR-1269a and miR-3179 mimics into 293T cells. **c** Four groups of 293T cells were transfected with different combinations of vectors (NC or miR-1269a mimic) and luciferase reporter plasmids (wild type or mutant type PCDHGA9). The luciferase activity was measured using the dual luciferase assay system. **d** The relative expression of PCDHGA9 was examined by qPCR following the transfection of miR-1269a inhibitor or mimic into CRC cells. **e** The protein levels of PCDHGA9 and β-catenin were analyzed by western blotting in CRC cells transfected with miR-1269a inhibitor or mimic. **f** The interacting region between PCDHGA9 and miR-1269a is shown

MicroRNAs have attracted substantial attention in recent tumor research owing to their pivotal roles, with numerous studies showing the association between dysregulation of oncogenes and microRNAs [23–25]. To further investigate the underlying cause for the reduced PCDHGA9 expression in colorectal tumors, we conducted a comprehensive analysis to predict the upstream regulatory microRNAs of PCDHGA9 using the StarBase, TargetScan, and miRBD bioinformatics tools, which identified hsa-miR-1269a and hsa-miR-3179 as common target microRNAs predicted by all three tools (Fig. 3a). However, our literature search showed that hsa-miR-5586-5p, hsa-miR-580-3p, hsa-miR-760, and hsa-miR-625-5p, which were identified as target microRNAs, demonstrated low expression in tumors or acted as inhibitors of cancer progression in previous studies [26–29]. This inconsistency with the anticipated goal of identifying highly expressed microRNAs that contribute to reduced PCDHGA9 expression and promote CRC progression prompted us to focus on miR-1269a and miR-3179. Then, we transfected miR-1269a and miR-3179 mimics into 293T cells, which revealed a robust downregulatory effect of miR-1269a on PCDHGA9, while miR-3179 exhibited a comparatively weaker effect on the downregulation of PCDHGA9 (Fig. 3b). Consequently, we selected miR-1269a as a potential upstream regulator of PCDHGA9 in the subsequent experiments.

HCT8 and SW620 cells were transfected with the miR-1269a mimic, whereas HCT116 and RKO cells were transfected with the miR-1269a inhibitor (Supplementary Fig. S2ab). Subsequently, we assessed the RNA and protein expression of PCDHGA9, revealing that miR-1269a exhibited a downregulatory effect on PCDHGA9 (Fig. 3d, e). To further validate the regulatory relationship between miR-1269a and PCDHGA9, luciferase assays were conducted in 293T cells. The results demonstrated that the miR-1269a mimic could effectively downregulate the luciferase expression of wild-type PCDHGA9 transcripts, which was nullified upon mutation of the predicted binding site, emphasizing the specificity of the interaction (Fig. 3c, f). These findings collectively confirm that miR-1269a exerts its influence by downregulating PCDHGA9 through the predicted binding site.

**Fig. 4:**
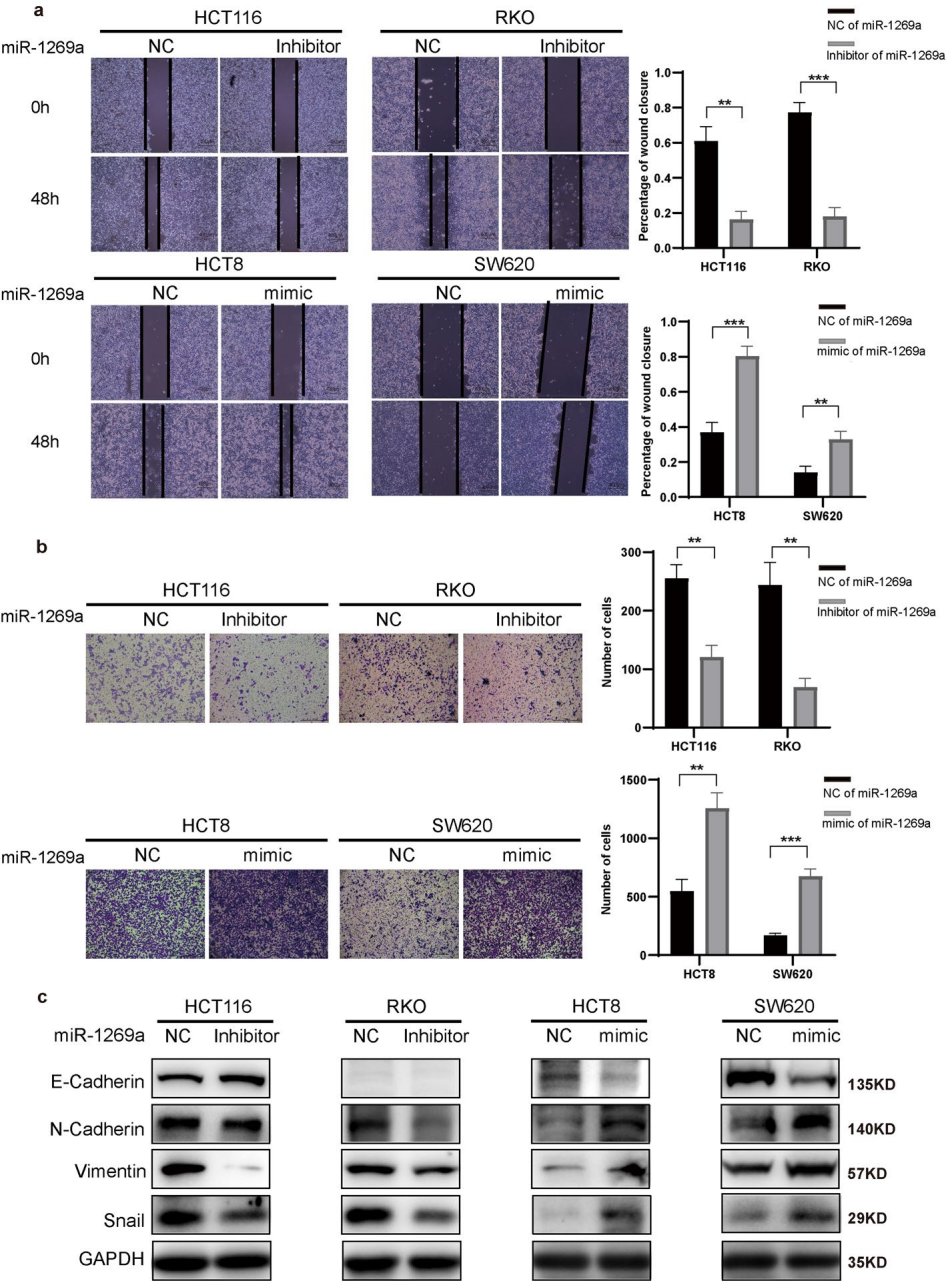
MiR-1269a promotes invasion, metastasis, and EMT of CRC cells. **a** Wound healing assays were conducted in CRC cells transfected with either miR-1269a inhibitor or mimic, along with their respective controls. The widths of wounds were measured at 0 h and 48 h, and the percentages of wound closure were calculated and analyzed. **b** Transwell assays were performed in CRC cells transfected with either miR-1269a inhibitor or mimic, along with their respective controls. The cells that had migrated out of the chamber were observed and counted. **c** Western blotting results demonstrated changes in the protein levels of E-cadherin, N-cadherin, vimentin, and Snail after transfection with either miR-1269a inhibitor or mimics into cells

Next, wound healing and Transwell assays were performed to detect the effects of miR-1269a on CRC cells’ invasion and metastasis ability, and the results showed that miR-1269a promoted the invasion and metastasis of CRC cells (Fig. 4a, b). Western blot analysis was conducted to evaluate EMT-related proteins and β-catenin expression, which showed that miR-1269a not only promoted the process of EMT in CRC cells but also led to an increase in β-catenin levels (Figs. 3e, 4c).

### CXCR4 is downregulated by PCDHGA9 interacting with HOXA1 and affects invasion, metastasis, and EMT of CRC cells

To further investigate the mechanism of β-catenin regulation by PCDHGA9, high-throughput sequencing results revealed a significant reduction in CXCR4 expression in cells overexpressing PCDHGA9. qRT-PCR analysis corroborated that both PCDHGA9 overexpression and knockdown led to corresponding changes in CXCR4 expression (Supplementary Fig. S2c, d). Considering that numerous studies have highlighted CXCR4’s role in inhibiting β-catenin degradation through the AKT/GSK-3β pathway, triggering intracellular β-catenin accumulation, Wnt pathway activation, and fostering cancer invasion and metastasis [30–32], we examined CXCR4 as a potential downstream target of PCDHGA9.

**Fig. 5:**
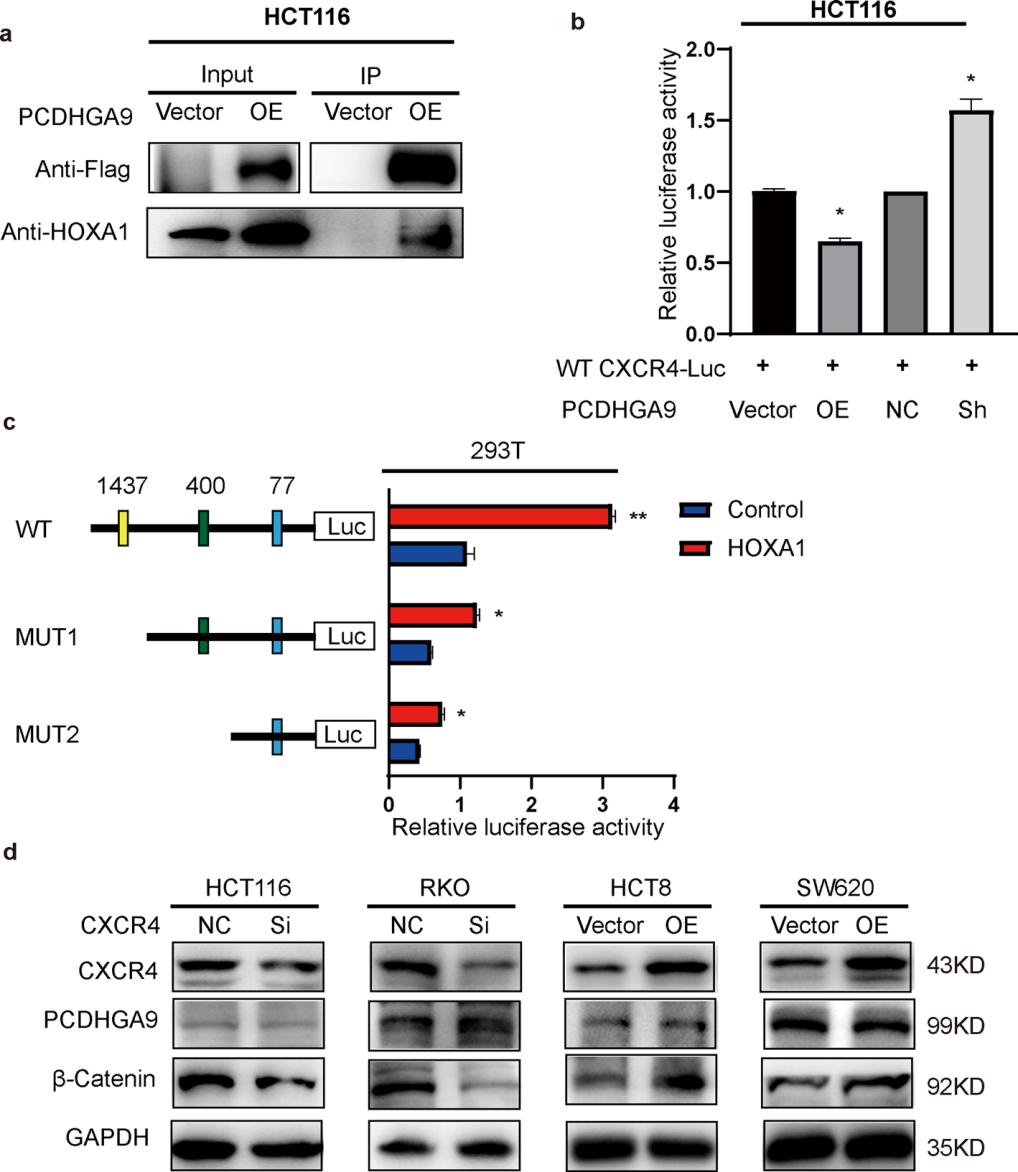
CXCR4 is downregulated by PCDHGA9 interacting with HOXA1. **a** Co-IP assays revealed the presence of the FLAG and HOXA1 proteins in both the input and immunoprecipitated samples. **b** Dual luciferase assays showing the relative activity of the wild-type (WT) CXCR4 luciferase reporter plasmid after either overexpressing or downregulating PCDHGA9. **c** Predicted binding sites of HOXA1 within the CXCR4 promoter region at positions 1437, 400, and 77. Two mutant constructs, MUT1 and MUT2, were generated for the three predicted binding sites. Dual luciferase assays were conducted to assess the relative activity of WT, MUT1, and MUT2 CXCR4 luciferase reporter plasmids in the presence or absence of HOXA1. **d** Western blot analysis depicting the protein expression of CXCR4, PCDHGA9, and β-catenin in CRC cells with altered CXCR4 expression

Recognizing that PCDHGA9 functions as a transmembrane protein rather than a direct transcription factor, its capacity for gene transcription is limited. On the basis of the interaction potential of the calmodulin protein family, we explored their influence on intracellular distribution and functionality. Through coimmunoprecipitation assay, we identified coprecipitated proteins in overexpressing PCDHGA9 cells, including transcription factors HOXA1, HOXB3, and HOXB13 (Supplementary Fig. S2e). According to JASPAR prediction, HOXA1 exhibited multiple high-affinity binding sites within the CXCR4 promoter region (Supplementary Fig. S2f), which reveals HOXA1 as an intermediary in the PCDHGA9–CXCR4 nexus. Western blot analysis confirmed PCDHGA9’s interactions with HOXA1, revealing its role in modulating HOXA1 distribution, particularly increasing cytoplasmic levels while reducing nuclear presence (Figs. 5a, 7c, d). The overexpression and knockdown of PCDHGA9 decreased and increased the activity of a luciferase plasmid driven by the CXCR4 promoter sequence, respectively (Fig. 5b). These results show the potential of PCDHGA9 in modulating the distribution of HOXA1, thereby influencing CXCR4 expression through the regulation of CXCR4 transcription.

To confirm HOXA1’s role as a CXCR4 transcription factor, we introduced a plasmid containing the HOXA1 transcript into HCT116 cells, which resulted in increased CXCR4 expression (Fig. 7b). Further, we created two mutations (MUT1 and MUT2) within the three predicted binding sites (Fig. 5c) and introduced them along with vector and HOXA1 overexpression. The results showed that HOXA1 overexpression enhanced CXCR4 expression across the three groups. However, when the CXCR4 promoter was mutated, the increase caused by HOXA1 was reduced (Fig. 5c), demonstrating that HOXA1 acts as a transcription factor for CXCR4, promoting its expression.

Combined with the above experiments, our analysis reveals that PCDHGA9 influences CXCR4 expression by interacting with HOXA1, a transcription factor for CXCR4, and influencing the intracellular distribution of HOXA1.

**Fig. 6:**
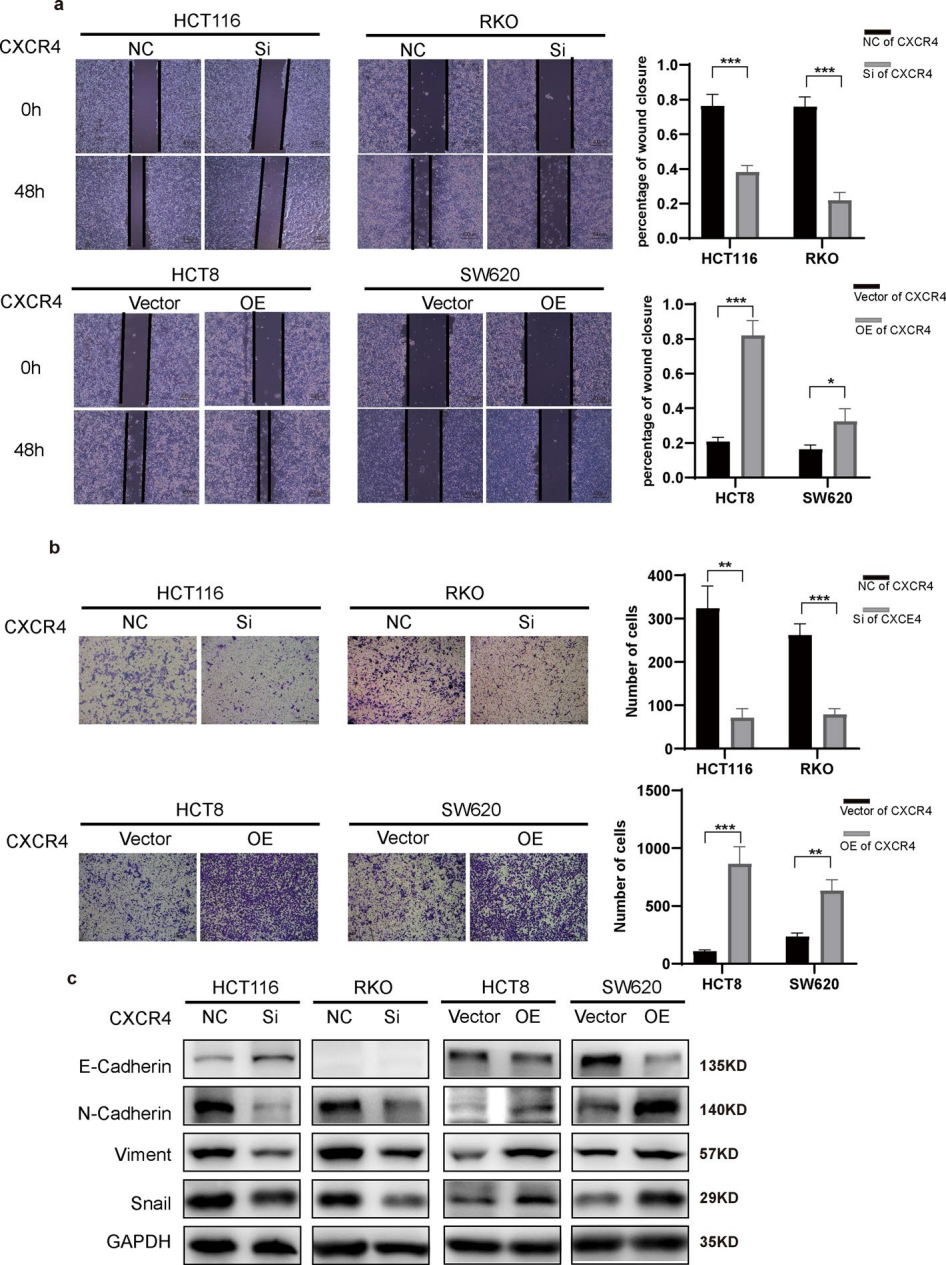
CXCR4 promotes invasion, metastasis, and EMT of CRC cells. Wound healing (**a**), Transwell assays (**b**), and WB (**c**) showing the effects of CXCR4 on promoting the invasion, metastasis, and EMT of CRC cells

Next, we examined the impact of CXCR4 on the invasive and metastatic abilities of CRC cells. The results showed that CXCR4 promotes invasion and metastasis (Fig. 6a, b), stimulates EMT, and increases β-catenin levels in CRC cells (Figs. 5d, 6c). Importantly, it was observed that CXCR4 did not influence PCDHGA9 (Fig. 5d).

### The regulatory relationship among PCDHGA9, miR-1269a, CXCR4, and β-catenin

**Fig. 7:**
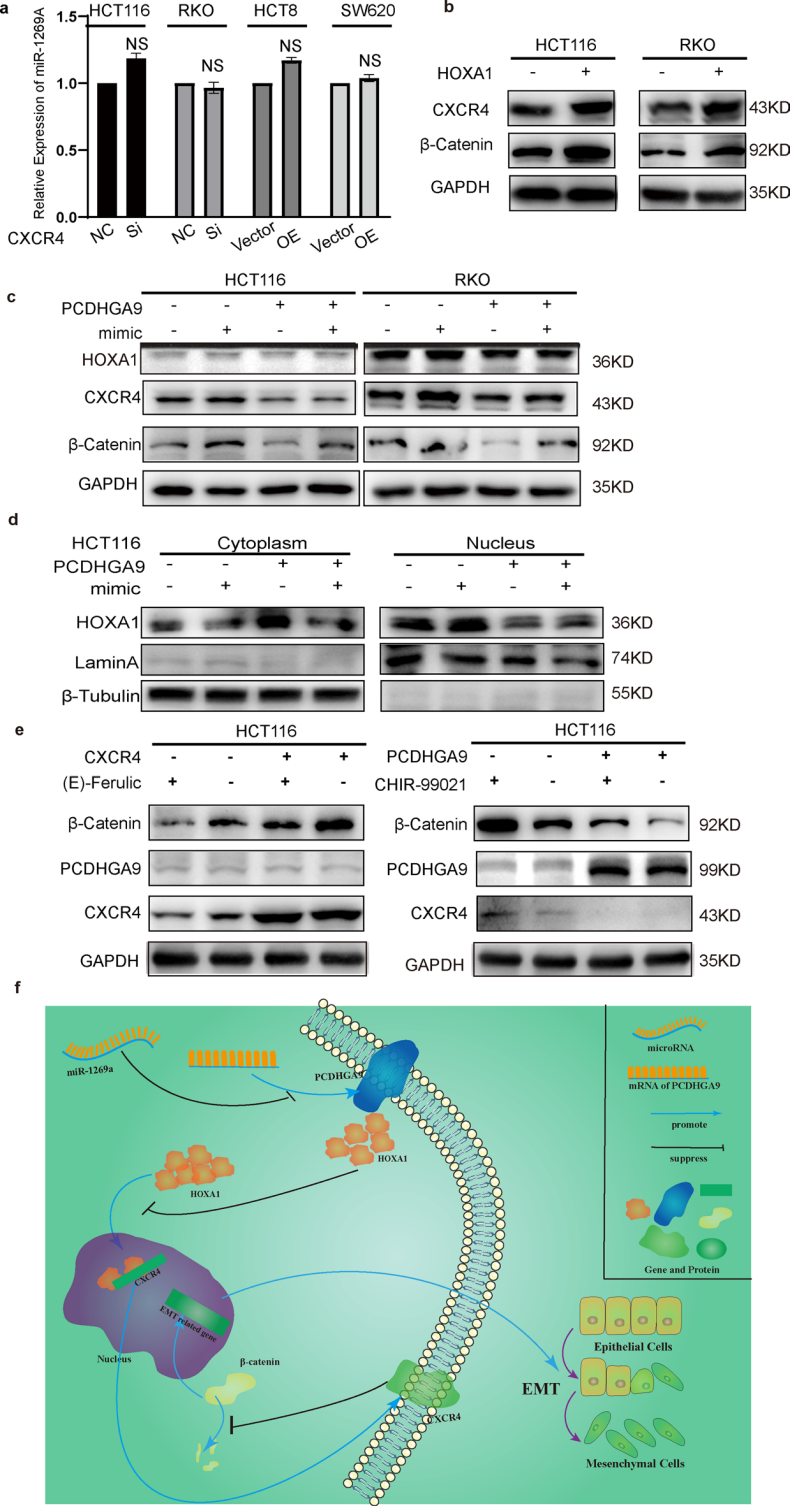
The regulatory relationship among PCDHGA9, miR-1269a, CXCR4, and β-catenin. **a** Quantitative PCR (qPCR) analysis demonstrated the expression levels of miR-1269a following the downregulation or overexpression of CXCR4. **b** Western blotting (WB) revealed the protein expression of CXCR4 and β-catenin in the presence or absence of the HOXA1 overexpressing plasmid. **c** WB showed alterations in the protein levels of HOXA1, CXCR4, and β-catenin upon PCDHGA9 overexpression and the introduction of the miR-1269a mimic. **d** WB depicted changes in the HOXA1 distribution between the cytoplasm and nucleus in the presence or absence of PCDHGA9 overexpression and the miR-1269a mimic. **e** WB analysis demonstrated that (*E*)-ferulic acid, a β-catenin inhibitor, affected β-catenin expression without impacting PCDHGA9 and CXCR4. Similarly, CHIR-99021, a β-catenin activator, influenced β-catenin expression while leaving PCDHGA9 and CXCR4 unaffected.** f** A schematic diagram illustrating the regulatory network involving miR-1269a, PCDHGA9, HOXA1, CXCR4, and β-catenin

We conducted retrospective experiments to confirm the interplay among PCDHGA9, miR-1269a, CXCR4, and β-catenin. Previous assays established that miR-1269a downregulates PCDHGA9, which then downregulates CXCR4 to affect β-catenin levels in CRC cells. However, altering PCDHGA9 expression did not affect miR-1269a levels when overexpressed or knocked down (Supplementary Fig. S1d). Similarly, CXCR4 had no impact on PCDHGA9 or miR-1269a (Figs. 5d, 7a). Then, we assessed their combined impact on downstream factors and found that miR-1269a downregulated both CXCR4 and β-catenin, whereas PCDHGA9 counteracted this downregulation (Fig. 7c). Moreover, miR-1269a counteracted the PCDHGA9-mediated transfer of HOXA1 from the nucleus to the cytoplasm (Fig. 7d). Next, we introduced (*E*)-ferulic acid, a β-catenin inhibitor, to vector and overexpressing CXCR4 cells and observed that (E)-ferulic acid impeded Wnt/β-catenin pathway activation driven by CXCR4 overexpression without significantly affecting miR-1269a, PCDHGA9, or CXCR4 expression (Fig. 7e, Supplementary Fig. S3a). Similarly, CHIR-99021, a β-catenin activator, was introduced to vector and overexpressing PCDHGA9 cells, which then counteracted the β-catenin reduction induced by PCDHGA9 while showing no substantial effect on miR-1269a, PCDHGA9, or CXCR4 (Fig. 7e, Supplementary Fig. S3b). Collectively, these findings suggest that β-catenin functions as a downstream regulatory target of miR-1269a, PCDHGA9, and CXCR4, which collectively constitute the miR-1269a/PCDHGA9/CXCR4/β-catenin pathway, which can be targeted to prevent CRC invasion and metastasis (Fig. 7f).

### miR-1269a and PCDHGA9 affect the invasion and metastasis of CRC

**Fig. 8:**
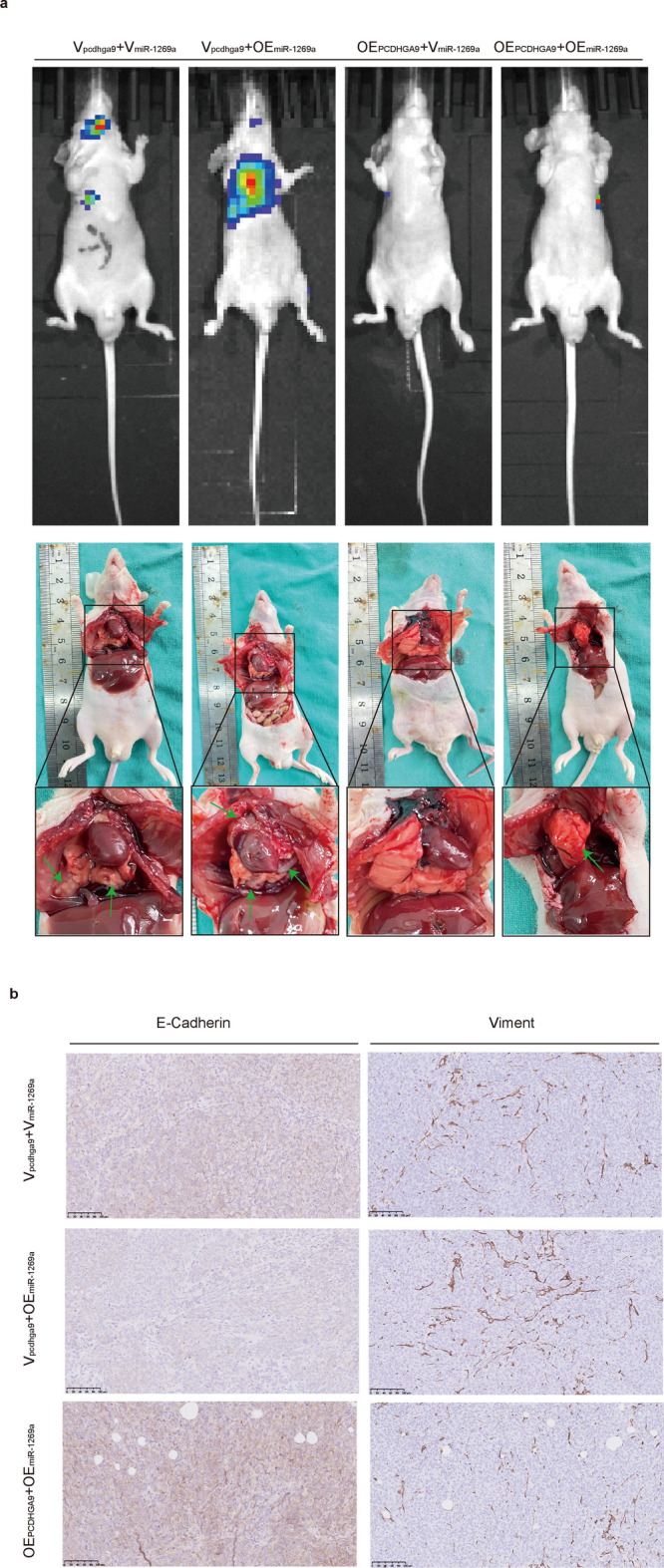
miR-1269a and PCDHGA9 together affect the invasion and metastasis of CRC in vivo. **a** Whole-body fluorescence images showing lung metastases across varying conditions of miR-1269a and PCDHGA9 expression. Subsequent photos depicted lung metastases following the sacrifice of these mice. **b** Immunohistochemical staining of lung metastases was carried out for E-cadherin and vimentin. The immunohistochemical staining highlighted alterations in E-cadherin and vimentin expression in response to different miR-1269a and PCDHGA9 expression conditions. miR-1269a reduced the expression of E-cadherin, but this result was reversed in the condition of high expression of PCDHGA9. Vimentin showed the opposite of E-cadherin

To investigate the combined impact of miR-1269a and PCDHGA9 on invasion and metastasis of CRC in vivo, we cotransfected two lentiviruses—V_pcdhga9_ or OE_pcdhga9_ and V_miR-1269a_ or OE_miR-1269a_—into HCT116 cells. Stable cotransfected cells were screened using puromycin and blasticidin. Subsequently, four groups of cells were used to establish lung metastasis models in nude mice. Whole-body fluorescence images revealed that the miR-1269a-only group, devoid of PCDHGA9, exhibited larger and more focal areas of metastatic shadows than the other groups, indicating that the miR-1269a-only group displayed increased invasive and metastatic abilities. Conversely, the group coexpressing miR-1269a and PCDHGA9 demonstrated reduced invasive and metastatic abilities compared with the miR-1269a-only group (Fig. 8a). However, no metastatic shadows were observed in the PCDHGA9-only group (Fig. 8a). Subsequent sacrifice of the mice for lung nodule assessment confirmed that the miR-1269a-only group had more nodules, while PCDHGA9 attenuated miR-1269a’s impact on nodule formation (Fig. 8a, Supplementary Fig. S3c). Similar results were observed in these nodules following immunohistochemical staining for E-cadherin and vimentin (Fig. 8b).

Moreover, we used clinical specimens to establish a combined molecular prognostic model involving miR-1269a and PCDHGA9 and evaluated the expression of miR-1269a in 63 colorectal cancer patient tissues (Supplementary Fig. S3d). However, Spearman’s correlation method revealed no significant correlation between the expressions of miR-1269a and PCDHGA9 in CRC tissues (Supplementary Fig. S3e).

## Discussion

With the growing utilization of electronic colonoscopy for routine examinations, an increase in early-stage CRC diagnosis has been observed [33]. Surgical interventions for these patients often result in favorable disease-free survival outcomes [34]. However, addressing the persistently low survival rates observed in advanced-stage CRC remains a challenge in modern cancer treatment [35]. Despite the implementation of precise minimally invasive surgical techniques, diverse chemotherapy regimens, and combined immunotherapies, the prevention of tumor recurrence remains difficult [36], which is particularly pronounced for CRC patients with tumors that have invaded through the serosa layer of the colon and distant organ metastases, as their 5-year survival rate remains at a mere 13.1% [34]. Despite extensive research into the mechanisms of tumor EMT regarding invasion and metastasis, a clinically applicable target capable of substantially altering the survival prospects of late-stage CRC patients has yet to be identified [3].

In this study, we investigated the impact of PCDHGA9 on the invasion and metastasis abilities of CRC on the basis of our previously identified functional characteristics in gastric cancer. Our findings reveal that PCDHGA9 inhibits the EMT of CRC by affecting the Wnt/β-catenin pathway through an alternative pathway and is an independent prognostic biomarker for CRC.

On the basis of clinical tissue expression analysis and pathological data, we observed lower PCDHGA9 expression in CRC tissues compared with adjacent normal tissues, which was correlated with more advanced pathological staging. Additionally, clustering analysis of differentially expressed genes indicated an association of PCDHGA9 with cellular adhesion functions in CRC, which led us to hypothesize a link between PCDHGA9 and CRC invasion and metastasis. Thus, we conducted Transwell and wound healing assays, which showed that, contrary to findings in our previous gastric cancer research, the overexpression of PCDHGA9 in CRC cells resulted in decreased expression of β-catenin.

Further examination of differentially expressed genes through high-throughput sequencing revealed a significant reduction in CXCR4 expression within overexpressed PCDHGA9 HCT116 cells. Previous studies have indicated that CXCR4 impedes the degradation of β-catenin, thus leading to the activation of the Wnt pathway. Consequently, CXCR4 was selected for subsequent investigation as a downstream element influenced by PCDHGA9. Through protein mass spectrometry analysis and the transcription factor prediction tool JASPAR, a potential interaction between HOXA1 and PCDHGA9 was identified, along with several predicted binding sites linking HOXA1 to the transcription start site of CXCR4. The regulatory relationship between PCDHGA9 and CXCR4 was verified through Co-IP, nuclear–cytoplasm separation, and dual luciferase assays. Specifically, PCDHGA9 interaction with HOXA1 caused HOXA1 to accumulate in the cytoplasm, restricting its nuclear entry, consequently inhibiting CXCR4 transcription and leading to β-catenin accumulation. Next, we found that miR-1269a negatively regulates the expression of PCDHGA9, thereby facilitating the invasion and metastasis of CRC. Thus, a regulatory pathway of miR-1269a/PCDHGA9/CXCR4/β-catenin was identified to modulate the EMT process in CRC.

There are some limitations to this study. Our objective was to enable more accurate clinical prognosis through the combined miR-1269a and PCDHGA9 dual-factor survival analysis. However, no significant correlation was observed between miR-1269a and PCDHGA9 in tissue expression. Several reasons could account for these findings. First, in vitro cell experiments failed to replicate miR-1269a’s tissue expression level. In PCR experiments, the cycle threshold (CT) value of miR-1269a in the in vitro cell NC group was over ten units higher than that in the mimic group, indicating significantly more miR-1269a in the mimic group compared with the NC group. Second, while the in vitro cell experiment verified miR-1269a’s negative regulation of PCDHGA9, the expression level of miR-1269a in CRC tissue was very low, with a CT value exceeding 30. Third, miR-1269a may have not been the only PCDHGA9 regulator in CRC; hence, the intricate molecular network within CRC cells might have obscured the miR-1269a and PCDHGA9 expression correlation. Another limitation was the absence of metastasis nodules in the overexpression of PCDHGA9 and vector of miR-1269a group. However, this may not imply that PCDHGA9 overexpression compromised CRC’s invasion and metastasis ability. It was speculated that using two cell toxins, puromycin and blasticidin, for screening could impact CRC cell viability. Additionally, PCDHGA9 overexpression reduced CRC cell invasion and metastasis ability, making metastatic nodule acquisition challenging. Regarding the differential effect of PCDHGA9 on the Wnt/β-catenin pathway between CRC and gastric cancers, varying HOXA1 and CXCR4 expression levels in these cancers might explain this difference. Similarly, this study observed varied degrees of CXCR4 recovery following the cooverexpression of PCDHGA9 and miR-1269a in HCT116 and RKO compared with PCDHGA9 and vector 2, which could be attributed to the different CXCR4 levels in HCT116 and RKO.

Tumor development and progression can be attributed significantly to the dysregulated expression of molecules within tumors [37]. β-Catenin holds a pivotal role in the Wnt pathway, where its cellular levels determine the activation or suppression of the pathway. In the canonical Wnt signaling cascade, β-catenin undergoes phosphorylation, ubiquitination, and subsequent degradation through protein complexes. This maintains intracellular β-catenin at a relatively low level to preserve cellular function at a physiological state [16]. However, the equilibrium between β-catenin production and degradation is disrupted in tumor cells, leading to its accumulation, which triggers a series of functions, including proliferation, invasion, metastasis, autophagy, and drug resistance [38]. One of the reasons for disrupting this balance is the genetic mutations upstream of β-catenin. The adenomatous polyposis coli (APC) gene, a well-known tumor suppressor, is a component of protein complexes, and somatic APC mutations are found in more than 80% of sporadic CRC [39]. However, the aggregation of β-catenin also activates other signaling pathways. For instance, β-catenin interacts with members of the Notch pathway, which are significant regulators of cell differentiation and play an important role in the carcinogenesis process of CRC [40]. Overall, our analysis of the miR-1269a/PCDHGA9/CXCR4/β-catenin signaling pathway has contributed to a deeper understanding of the broader Wnt/β-catenin pathway network.

Despite advancements in precise minimally invasive surgical techniques, diverse chemotherapy regimens, and combined immunotherapies, tumor recurrence remains challenging to prevent, especially for CRC patients with tumors that have invaded the serosa layer of the colon and distant organ metastases. Unfortunately, their five-year survival rate remains low at 13.1% [41]. Currently, surgical resection is the only possible means of achieving a cure. While accomplishing en bloc resection of invaded structures to attain clear surgical margins (R0 resection) in stage I, II, and III of CRC is relatively feasible, the role of surgery in treating metastatic CRC is currently a topic of considerable debate [42]. Therefore, molecular drug therapy has assumed a more prominent role in addressing metastatic CRC. Our exploration of the miR-1269a/PCDHGA9/CXCR4/β-catenin signaling pathway holds promise as it offers potential targets and strategies for pharmacotherapy in CRC.

## Conclusions

PCDHGA9 acts as a tumor suppressor with anti-invasive and metastatic ability in CRC. miR-1269a is one of the upstream regulators of PCDHGA9, which could downregulate PCDHGA9 and promote the invasion, metastasis, and EMT of CRC by the miR-1269a/PCDHGA9/CXCR4/β-catenin signaling pathway. And the reduction of PCDHGA9 can serve as an independent prognostic biomarker in CRC.

## Supplementary Information

Supplementary Material 1.

## Data Availability

All data and materials are available in this article. If you want some further details, please contact the corresponding author with a reasonable request.

## Abbreviations

CRC: Colorectal cancer
PCDHGA9: Protocadherin gamma subfamily A, 9
HOXA1: Homeobox A1
HOXB3: Homeobox B3
HOXB13: Homeobox B13
CXCR4: C–X–C motif chemokine receptor 4
OE: Overexpression
NC: Negative control
Sh: Short hairpin
Si: Small interfering
EMT: Epithelial–mesenchymal transition
RT-qPCR: Real-time quantitative polymerase chain reaction
IPA: Ingenuity pathway analysis
APC: Adenomatous polyposis coli
TCF: T-cell-specific factor
LEF: Lymphoid enhancer-binding factor
Co-IP: Coimmunoprecipitation
WT: Wild type
MUT1: Mutation 1
MUT2: Mutation 2
CT: Cycle threshold
