# Correction: The miR-1269a/PCDHGA9/CXCR4/β-catenin pathway promotes colorectal cancer invasion and metastasis

**DOI:** 10.1186/s11658-025-00688-9

**Published:** 2025-01-31

**Authors:** Haitao Mei, Qingshan Luo, Junyong Weng, Jialing Hao, Jinfeng Cai, Runkai Zhou, Ce Bian, Yingzi Ye, Shengzheng Luo, Yugang Wen

**Affiliations:** 1 Department of Gastrointestinal Surgery, Shanghai Municipal Hospital of Traditional Chinese Medicine, Shanghai University of Traditional Chinese Medicine, 274 Zhijiang Middle Road, Shanghai, 200071 China; 2 Department of General Surgery, Shanghai General Hospital, Shanghai Jiao Tong University School of Medicine, 85 Wujin Road, Shanghai, 200080 China; 3 Department of Colorectal Surgery, Changzheng Hospital, Navy Medical University, 415 Fengyang Road, Shanghai, 200003 China; 4 Department of Gastroenterology, Shanghai General Hospital, Shanghai Jiao Tong University School of Medicine, 85 Wujin Road, Shanghai, 200080 China; 5 Department of Colorectal Surgery, Fudan University Shanghai Cancer Center, 270 Dong’an Road, Shanghai, 200032 China; 6 Department of Infectious Diseases, Children’s Hospital of Fudan University, 399 Wanyuan Road, Shanghai, 201102 China

**Correction: Cellular & Molecular Biology Letters (2024) 29:144** 10.1186/s11658-024-00656-9

Following publication of the original article [1], the authors identified an error in the *Correspondence section and in the author names of Yingzi Ye, Shengzheng Luo and Yugang Wen

The incorrect author names are: Ye Yingzi, Luo Shengzheng and Wen Yugang

The correct author names are: Yingzi Ye, Shengzheng Luo and Yugang Wen

The *Correspondence section and the author names have been updated above and the original article has been corrected.
