# Supplementary material for: The miR-1269a/PCDHGA9/CXCR4/β-catenin pathway promotes colorectal cancer invasion and metastasis
Source: Cell Mol Biol Lett. 2024 Nov 26;29:144. doi: 10.1186/s11658-024-00656-9 (PMC11590219; doi:10.1186/s11658-024-00656-9)
Supplement: Supplementary file 1 — Supplementary Material 1. [file 11658_2024_656_MOESM1_ESM.docx]

**
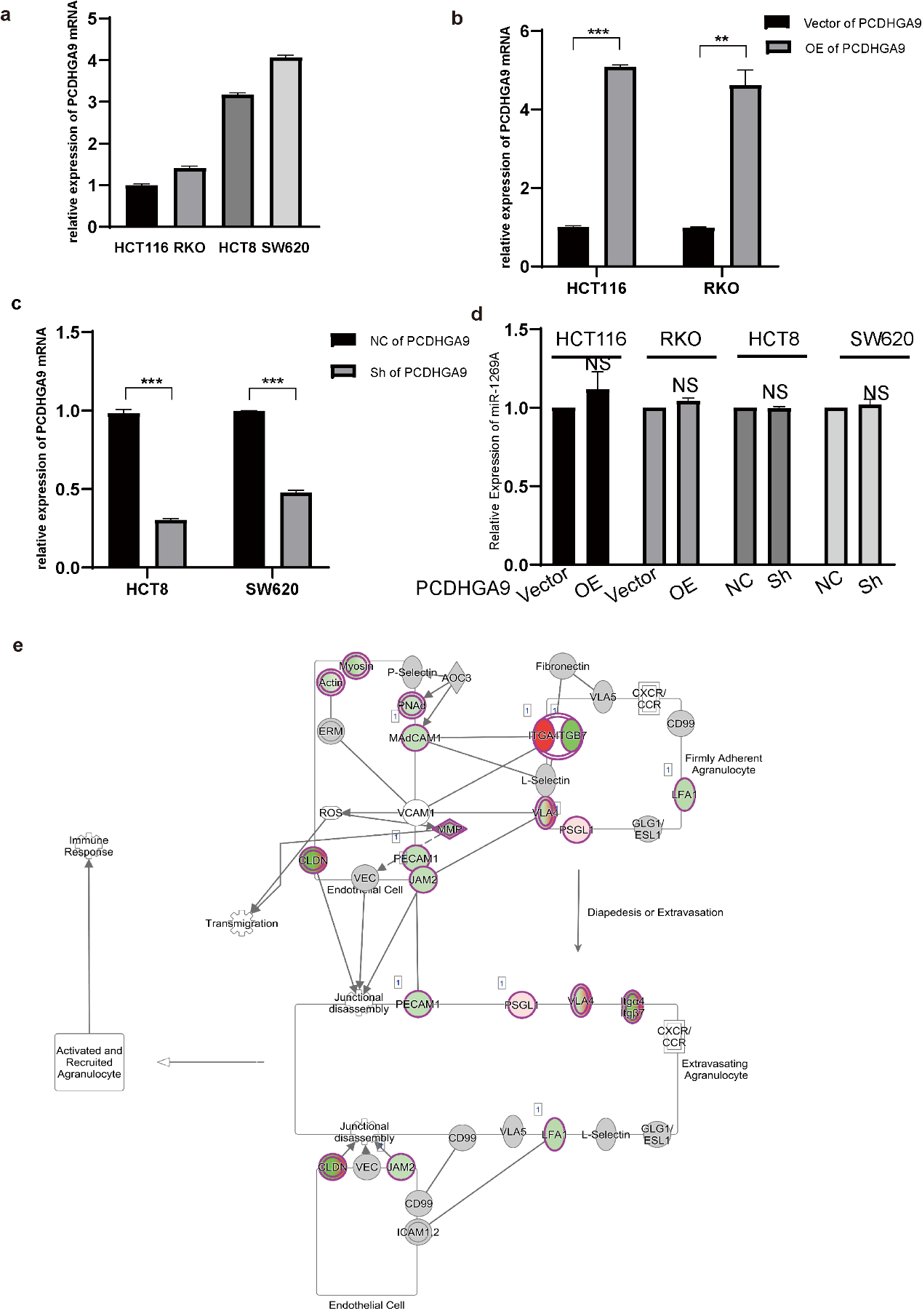
**

**Figure S1**.

**a** the PCDHGA9 mRNA expression levels in HCT116, RKO, HCT8, SW620. **bc** qPCR results showing the relative expression of PCDHGA9 mRNA in response to PCDHGA9 overexpression or downregulation.**d** Relative expression of miR-1269a have no significant difference when PCDHGA9 is overexpressed or downregulated in HCT116, RKO, HCT8 and SW620 cell lines. **e** Ingenuity Pathway Analysis (IPA) showing the relative biological functions of PCDHGA9 using high-throughput sequencing data from differentially expressed genes.


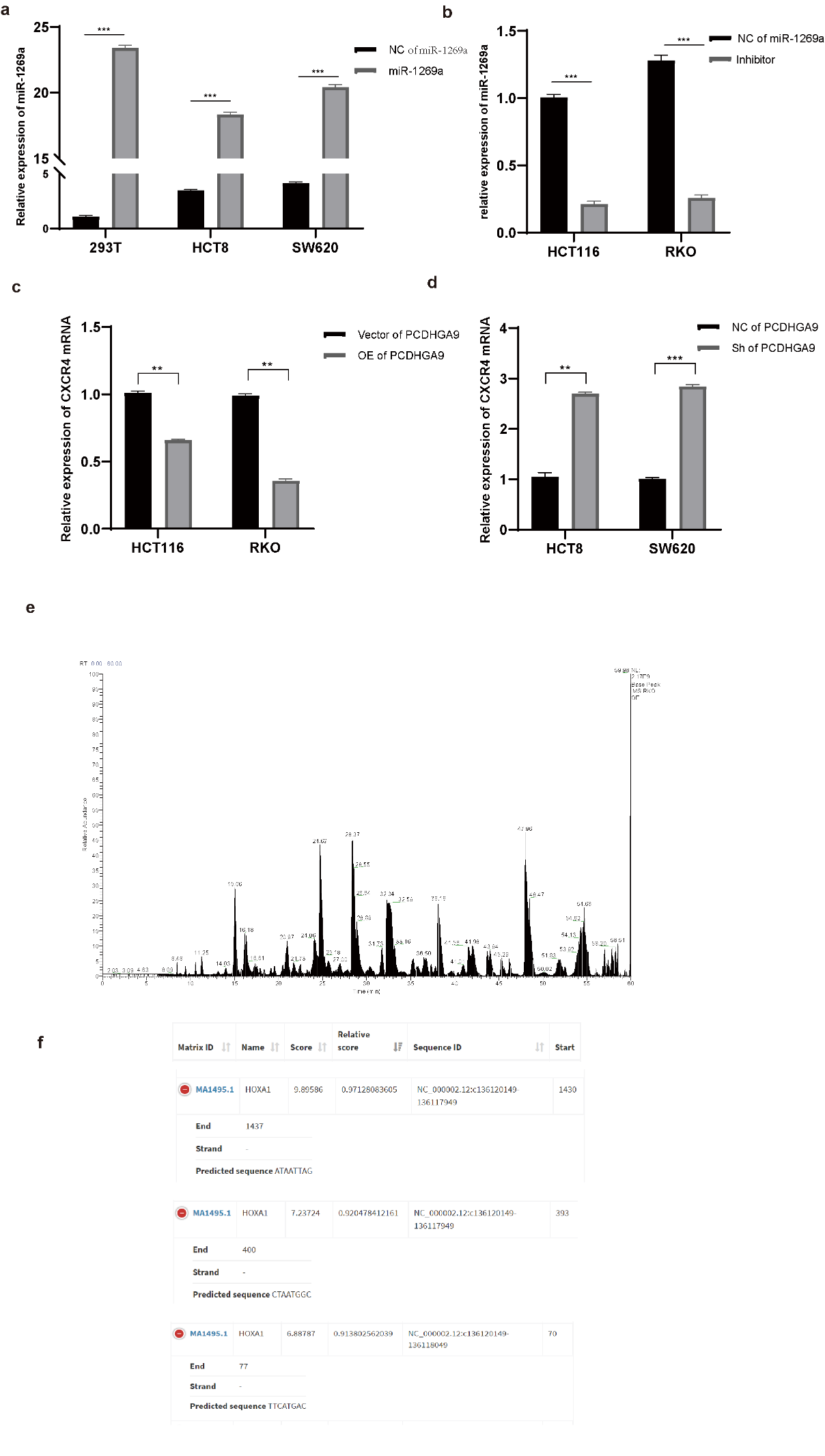


**Figure S2.**

**ab** qPCR analysis showing the relative expression of miR-1269a upon transfection of miR-1269a mimic or inhibitor in CRC cells. **cd** qPCR results depicting the relative expression of CXCR4 in response to PCDHGA9 overexpression or downregulation. **e** Protein Mass Spectrometry was conducted on proteins pulled down by PCDHGA9, revealing transcription factor proteins, including HOXA1, HOXB3, and HOXB13 among the co-precipitated proteins. **f** JASPAR predicted three binding sites of HOXA1 with the promoter sequence of CXCR4.


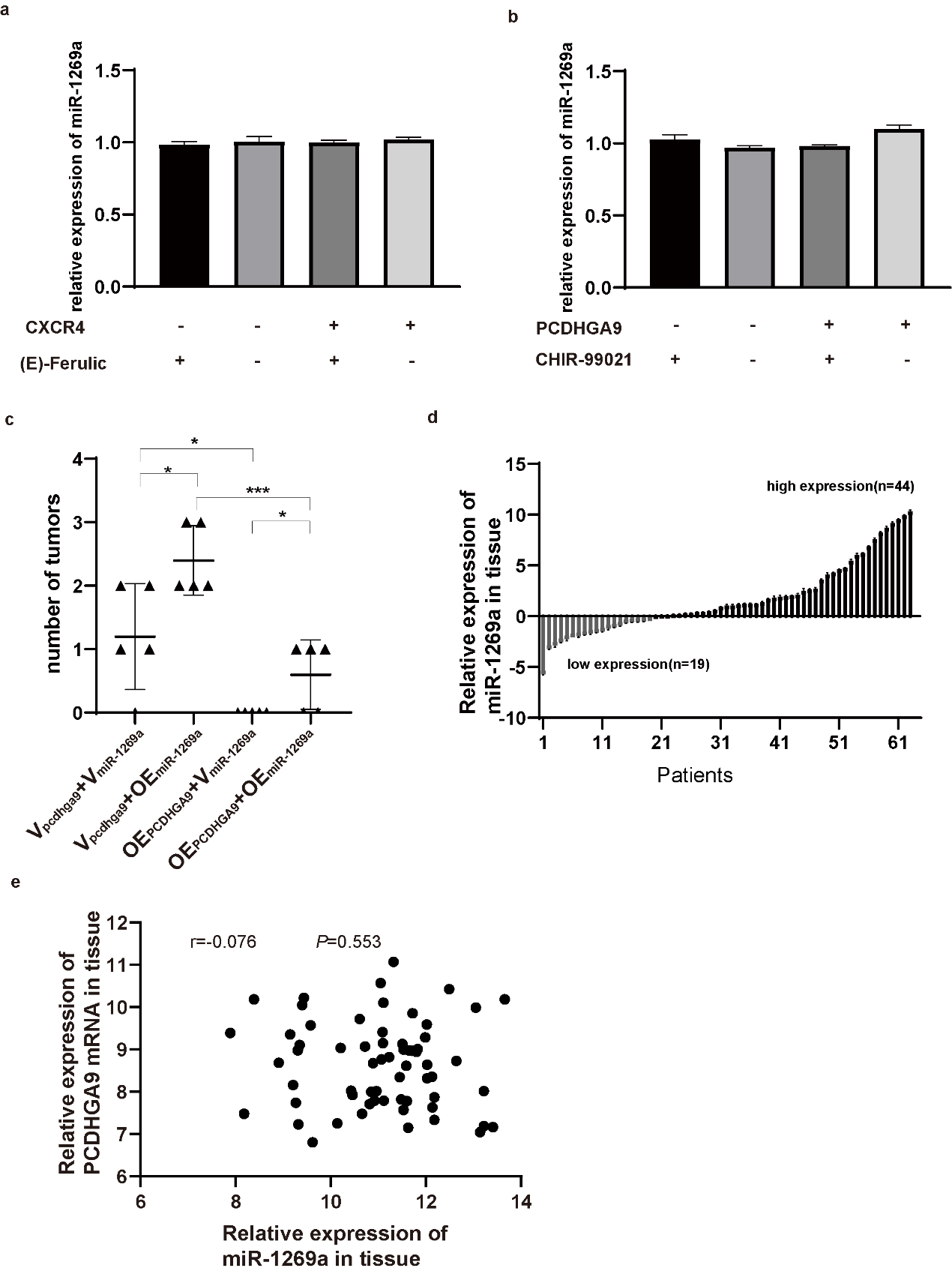


**Figure S3.**

**ab** The expression of miR-1269a showed minimal variation with changes in PCDHGA9, CXCR4, and β-Catenin. **c** The count of lung metastatic tumors quantified across diverse miR-1269a and PCDHGA9 expression conditions. **d** qPCR analysis demonstrating the expression of miR-1269a across 63 CRC tissues. **e** Spearman's correlation analysis revealed no significant correlation between the expression of miR-1269a and PCDHGA9 in CRC tissues (r=-0.076, *P*=0.553).
